# Anaemia, idiopathic intracranial hypertension (IIH) and spaceflight associated neuro-ocular syndrome (SANS)

**DOI:** 10.1038/s41433-023-02834-z

**Published:** 2023-12-02

**Authors:** Ethan Waisberg, Joshua Ong, Andrew G. Lee

**Affiliations:** 1 Department of Ophthalmology, University of Cambridge, Cambridge, UK; 2 Michigan Medicine, University of Michigan, Ann Arbor, MI USA; 3 Center for Space Medicine, Baylor College of Medicine, Houston, TX USA; 4 Department of Ophthalmology, Blanton Eye Institute, Houston Methodist Hospital, Houston, TX USA; 5 The Houston Methodist Research Institute, Houston Methodist Hospital, Houston, TX USA; 6 Departments of Ophthalmology, Neurology, and Neurosurgery, Weill Cornell Medicine, New York, NY USA; 7 Department of Ophthalmology, University of Texas Medical Branch, Galveston, TX USA; 8 University of Texas MD Anderson Cancer Center, Houston, TX USA; 9 Texas A&M College of Medicine, Bryan, TX USA; 10 Department of Ophthalmology, The University of Iowa Hospitals and Clinics, Iowa City, IA USA

**Keywords:** Pathogenesis, Diseases

With the commercialisation of spaceflight, the amount of space travellers will rise exponentially in the coming years. Alongside this, the prevalence of spaceflight associated neuro-ocular syndrome (SANS) will also likely increase. SANS serves as one of the largest physiological barriers to spaceflight, however currently the underlying pathophysiology of this syndrome is poorly defined. SANS is a collection of distinct neuro-ophthalmic findings seen in astronauts following long-duration spaceflight (LDSF) characterised by posterior globe flattening, optic disc oedema and chorioretinal folds. All of these finding may also be present in a terrestrial patient with IIH, however unlike IIH, SANS is not associated with pulsatile tinnitus, neck stiffness or double vision.

The aetiology of SANS remains unknown but it is hypothesised to be associated with the redistribution of fluids that occurs in microgravity [1, 2]. Ferguson et al. [3] recently compared the location and frequency of choroidal and retinal fold patterns in IIH and SANS and concluded that parallels exist, and that elevated ICP may play a role in the onset of SANS. Vosoughi et al [4] recently conducted a prospective study documenting that up to 1 in 5 patients with idiopathic intracranial hypertension (IIH) had anaemia with 10% of these patients harbouring a severe anaemia. While previous case reports [5], and systematic reviews [6] have previously suggested a possible association between anaemia and IIH, this prospective study with 143 patients makes this association even more compelling, while also providing a practical recommendation to perform a complete blood count for all suspected IIH patients.

SANS and IIH are both severe diseases for their different demographics, and further understanding the role of anaemia in these conditions is essential. There is a potential association between anaemia and spaceflight associated neuro-ocular syndrome (SANS) [7]. Space anaemia, refers to a decrease in the number of circulating red blood cells and plasma volume as a result of selective haemolysis that occurs during spaceflight [8]. This process occurs gradually, with the concentration of haemoglobin gradually decreasing over time [8, 9]. The inclusion of anaemia screening for astronauts and in future surveillance efforts may be useful in providing insights into the pathophysiology of SANS. Further research in this area may lead to mitigation efforts and countermeasures for SANS and terrestrial IIH.
